# *Listeria monocytogenes* Response to Propionate Is Differentially Modulated by Anaerobicity

**DOI:** 10.3390/pathogens7030060

**Published:** 2018-06-29

**Authors:** Erica Rinehart, Eric Newton, Megan A. Marasco, Kaitlin Beemiller, Ashley Zani, Melani K. Muratore, John Weis, Nicole Steinbicker, Nathan Wallace, Yvonne Sun

**Affiliations:** Department of Biology, University of Dayton, Dayton, OH 45469, USA; erinehart1@udayton.edu (E.R.); newtoeri000@gmail.com (E.N.); marascom1@udayton.edu (M.A.M.); beemiller.3@wright.edu (K.B.); ashley.zani@osumc.edu (A.Z.); muratorem1@udayton.edu (M.K.M.); weisj@udayton.edu (J.W.); steinbic14@gmail.com (N.S.); wallacen1@udayton.edu (N.W.)

**Keywords:** short chain fatty acids, listeriolysin O, adherent growth, membrane fatty acid composition

## Abstract

Propionate is a common food preservative and one of the major fermentation acids in the intestines. Therefore, exposure to propionate is frequent for foodborne pathogens and likely takes place under suboxic conditions. However, it is not clear whether the absence of oxygen affects how pathogens respond to propionate. Here, we investigated how propionate exposure affects *Listeria monocytogenes* growth and virulence factor production under aerobic or anaerobic conditions and showed that oxygen indeed plays a key role in modulating *L. monocytogenes* response to propionate. Under aerobic conditions, propionate supplementations had no effect on planktonic growth but resulted in decreased adherent growth. Under anaerobic conditions, propionate supplementations resulted in a pH-dependent inhibition of planktonic growth and increased adherent growth. Cultures grown with propionate accumulated higher levels of acetoin under aerobic conditions but lower levels of ethanol under both aerobic and anaerobic conditions. Metabolic perturbations by propionate were also evident by the increase in straight chain fatty acids. Finally, propionate supplementations resulted in increased listeriolyin O (LLO) production under anaerobic conditions but decreased LLO production under aerobic conditions. These results demonstrate for the first time that the presence or absence of oxygen plays a critical role in shaping *L. monocytogenes* responses to propionate.

## 1. Introduction

*Listeria monocytogenes* is a ubiquitous bacterium that frequently enters into human food supply chains and causes fatal infections. In fact, *L. monocytogenes* alone is responsible for approximately 19% of annual deaths caused by foodborne infections [1]. The high mortality rate of *L. monocytogenes* infections is attributed to the virulence of outbreak strains and the immunocompromised states of infected individuals. However, the ubiquity of *L. monocytogenes* is attributed to the unique ability of *L. monocytogenes* to survive and grow through common food preservation strategies such as refrigeration [2]. As a result, *L. monocytogenes* surveillance is a crucial approach to prevent exposure and, as a routine practice, often results in frequent and costly food recalls. Therefore, to ensure food safety, reducing *L. monocytogenes* contamination in the food production and distribution is a challenging but necessary action.

Propionate is a Generally Recognized As Safe (GRAS) antimicrobial and flavoring agent [3] and its antimicrobial function against *L. monocytogenes* is under active investigation. Among the variety of food products that have been tested, such as ready-to-eat meat [4,5,6], raw poultry [7], and produce [8,9], propionate exhibited anywhere from no effect [7,8] to a significant growth inhibitory effect [4,5]. While the exact conditions required for propionate to inhibit *L. monocytogenes* growth are not clear in food products, it was shown that the presence of propionate at concentrations as little as 4 mM could decrease *L. monocytogenes* in vitro growth in rich media [10,11]. The inhibitory effect was not a result of ionic strength [11] and was exacerbated at lower temperature [10]. Moreover, compared to no propionate controls, *L. monocytogenes* treated with propionate for 1 h exhibited reduced survival on agar plates supplemented with 6% NaCl as well as reduced lactate dehydrogenase activity in cell free extract [12]. These preliminary studies suggest a potentially temperature-dependent effect of propionate on *L. monocytogenes* stress response as well as carbon metabolism.

In addition to being a common food additive, propionate is one of the main short chain fatty acids produced by the human gut microbiota and exhibits a wide range of regulatory and nutritional functions in human biology [13,14,15,16,17,18]. Foodborne pathogens are therefore routinely exposed to propionate during the intestinal phase of infection. However, relatively little is known about how enteric pathogens respond to propionate and whether propionate exposure alters the infection outcomes by different enteric pathogens. In *Salmonella*, propionate adaptation results in increased stress resistance but decreased mouse colonization [19,20]—a compromised virulence phenotype supported by a later observation that the metabolism of propionate to propionyl-CoA resulted in the decreased stability of the *Salmonella* virulence regulator HilD [21]. Whether *L. monocytogenes* virulence is regulated by propionate exposure or metabolism is completely unknown.

*L. monocytogenes* exposure to propionate can take place in food products and in the intestinal tract, during both of which *L. monocytogenes* may experience suboxic or anoxic conditions. Modified Atmospheric Packaging limits the amount of oxygen to reduce microbial growth and extend shelf life of food products [22]. The human gastrointestinal tract also exhibits spatial variations of oxygen saturation [23,24,25,26,27,28]. Therefore, it is important to consider the effects of anaerobic conditions on the response of *L. monocytogenes* to propionate to better establish the full impact of propionate exposure on *L. monocytogenes* behavior inside and outside of the host environment. A more in-depth understanding of how *L. monocytogenes* responds to propionate under both aerobic and anaerobic conditions will strengthen our ability to use propionate as an antimicrobial food additive and will enable us to explore propionate as a potential therapeutic or preventative agent against *L. monocytogenes* infections. In this study, we investigated the effects of propionate on *L. monocytogenes* growth and virulence factor production to obtain a better understanding of *L. monocytogenes* response to propionate under aerobic and anaerobic conditions.

## 2. Results

### 2.1. Propionate Perturbation on L. monocytogenes Planktonic Growth

**Figure 1:**
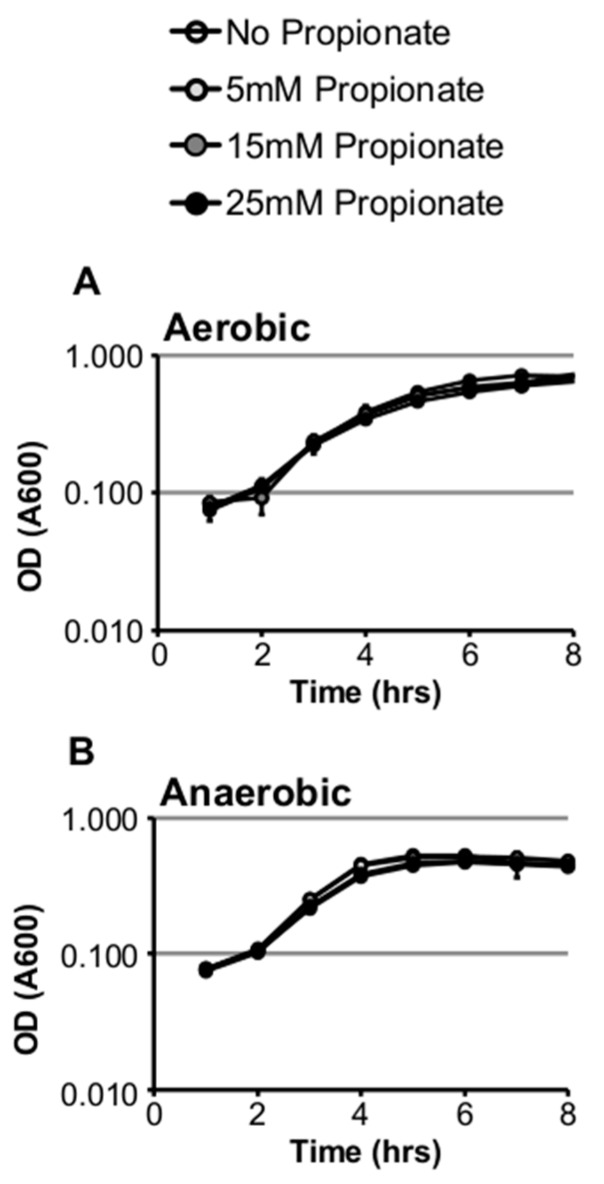
Supplementation of exogenous propionate did not inhibit *L. monocytogenes* planktonic growth in brain heart infusion (BHI) at 37 °C under aerobic (**A**) or anaerobic (**B**) conditions. Averages of triplicates were plotted with error bars representing standard deviations. Results represent three independent experiments.

**Figure 2:**
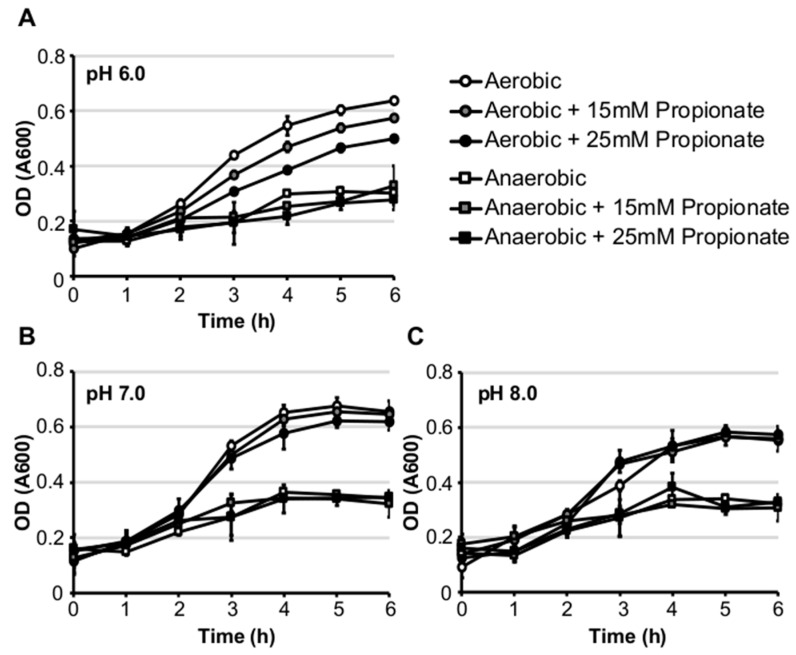
Supplementation of exogenous propionate resulted in reduced in vitro growth in BHI buffered at pH 6.0 under aerobic but not anaerobic conditions at 37 °C (**A**). In BHI buffered at pH 7.0 (**B**) or 8.0 (**C**), the inhibitory effects of propionate were alleviated. Averages of triplicates were plotted with error bars representing standard deviations. Results represent three independent experiments.

We first investigated the effect of propionate on in vitro growth of *Listeria monocytogenes* strain 10403s in standard brain heart infusion (BHI) medium under aerobic or anaerobic conditions. Supplementation of propionate at concentrations as high as 25 mM did not inhibit growth under aerobic or anaerobic conditions at 37 °C (Figure 1A,B). However, propionate supplementation at 25 mM resulted in a significant increase in doubling time under anaerobic but not aerobic conditions (Table 1). In contrast to growth at 37 °C, supplementation of propionate at room temperature resulted in a significant decrease in doubling time under aerobic but not anaerobic conditions (Table 1). We also tested the effects of propionate on *L. monocytogenes* growth in BHI buffered at pH 6.0, 7.0, or 8.0 and found a pH-dependent effects of propionate under aerobic but not anaerobic conditions. Under anaerobic conditions, growth in buffered BHI was not impacted by propionate supplementation. However, under aerobic conditions, growth was reduced by propionate supplementation in a dose-dependent manner most notably in BHI buffered at 6.0 (Figure 2A–C). These results suggest that the inhibitory effect of propionate on *L. monocytogenes* planktonic growth in BHI is heavily influenced by pH, temperature, and the presence or absence of oxygen.

### 2.2. Propionate Perturbation on L. monocytogenes Adherent Growth

**Figure 3:**
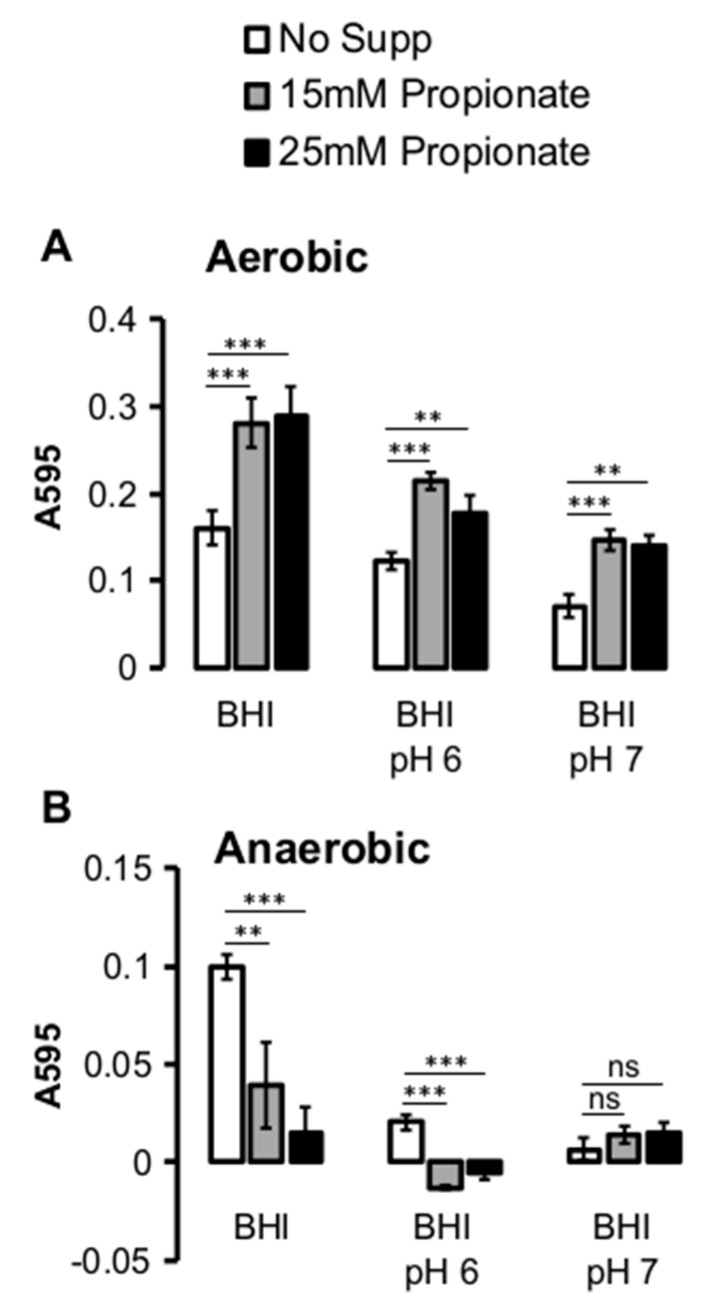
Supplementation of propionate altered *L. monocytogenes* adherent growth in polystyrene 96-well plates. (**A**) After 24 h of incubation, propionate supplementation resulted in an increase in the amount of adherent bacteria under aerobic conditions in non-buffered BHI (“BHI”) and BHI buffered at pH 6.0 (“BHI pH 6”) and pH 7.0 (“BHI pH 7”); (**B**) Under anaerobic conditions, propionate supplementation in non-buffered BHI resulted in a decrease in the amount of adherent bacteria. Little to no adherent bacteria were found in buffered BHI. Averages of five replicates per experiment were plotted with error bars representing standard deviations. Results represent three independent experiments. “**” indicates *p* values between 0.01 and 0.001 while “***” indicates *p* values less than 0.001. “NS” indicates sample pairs with no significant differences.

In contrast to planktonic growth, the effects of propionate on *L. monocytogenes* adherent growth were observed under both aerobic and anaerobic conditions. Under aerobic conditions, the presence of propionate resulted in an increase in the amount of adherent growth after 24 h of incubation, regardless of the type of media (Figure 3A). Under anaerobic conditions, there was generally much less adherent growth compared to that under aerobic conditions (Figure 3B). Extending the growth period to 48 h did not result in higher levels of adherent growth (data not shown). Under anaerobic conditions, the adherent growth was compromised in buffered BHI, regardless of the presence or absence of propionate. In non-buffered BHI, however, the presence of propionate resulted in a significantly reduced adherent growth. These results highlighted the effects of propionate on adherent growth and suggested a potential role of oxygen in modulating how propionate affects *L. monocytogenes* adherence to surfaces.

### 2.3. Propionate Perturbation on L. monocytogenes Metabolism

**Figure 4:**
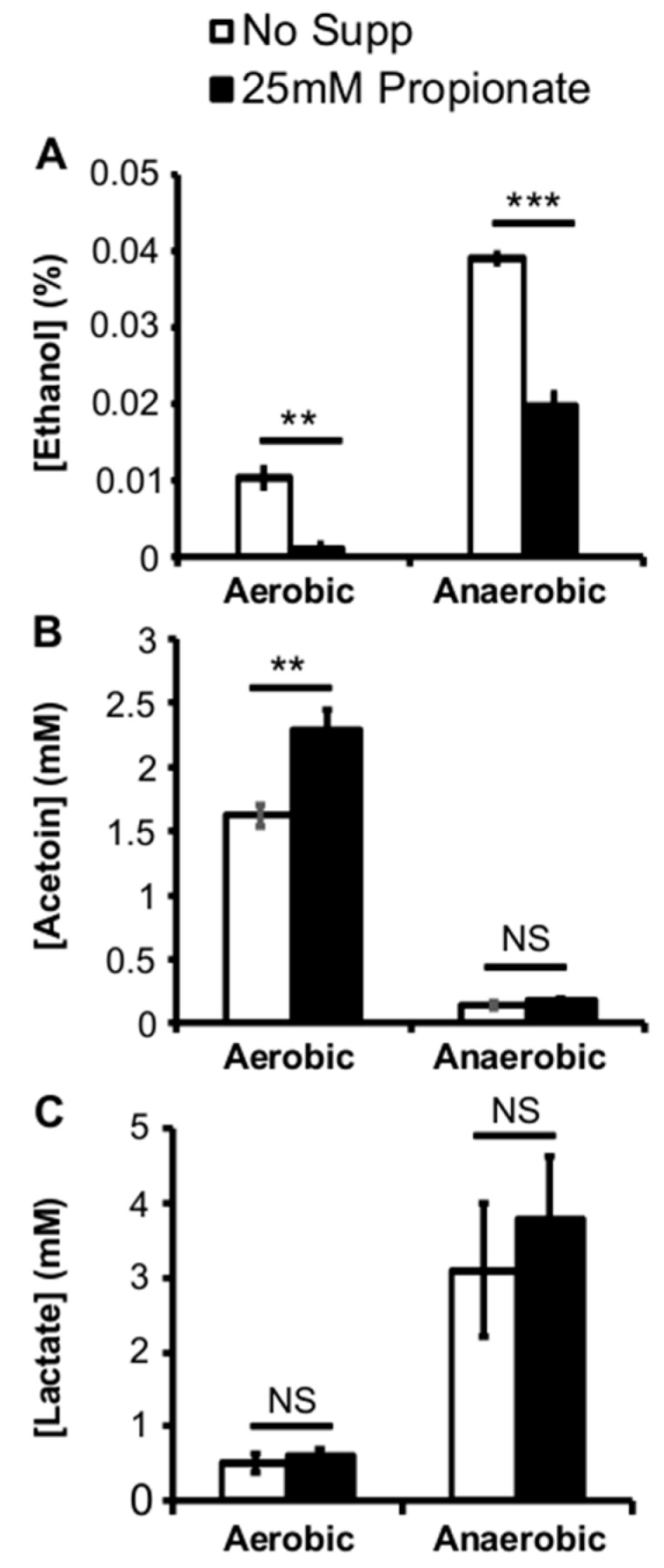
Supplementation of exogenous propionate at 25 mM resulted in reduced ethanol concentrations (**A**) in the supernatant of cultures grown under aerobic and anaerobic conditions; In contrast, supplementation of propionate resulted in increased acetoin concentrations only under aerobic conditions (**B**); Lactate concentrations in the culture supernatant were not affected by propionate supplementations (**C**). Averages of triplicates were plotted with error bars representing standard deviations. Results represent three independent experiments. “**” indicates *p* values between 0.01 and 0.001 while “***” indicates *p* values less than 0.001. “NS” indicates sample pairs with no significant differences.

To better understand how propionate affects *L. monocytogenes* growth, the culture pH was measured to examine the potential impact of propionate on acid production as an indirect indicator of metabolic activities. Interestingly, propionate supplementation resulted in a significant increase in culture pH under both aerobic and anaerobic conditions (Table 1). Because *L. monocytogenes* generates fermentation acids such as lactic acid as part of its carbon metabolism, the observed increase in culture pH in response to propionate may reflect an altered carbon metabolism. To determine whether propionate supplementation resulted in a shift in carbon metabolism, we further measured the concentrations of ethanol, acetoin, and lactate in the culture supernatant. The supplementation of propionate resulted in a significant decrease in the production of ethanol under both aerobic and anaerobic conditions (Figure 4A). In contrast, supplementation of propionate resulted in significantly increased production of acetoin under aerobic but not anaerobic conditions (Figure 4B). Supplementation of propionate did not alter the production of lactate (Figure 4C). These results suggest that exogenous propionate can influence *L. monocytogenes* central carbon metabolism. 

### 2.4. Propionate Perturbation on L. monocytogenes Fatty Acid Composition

**Figure 5:**
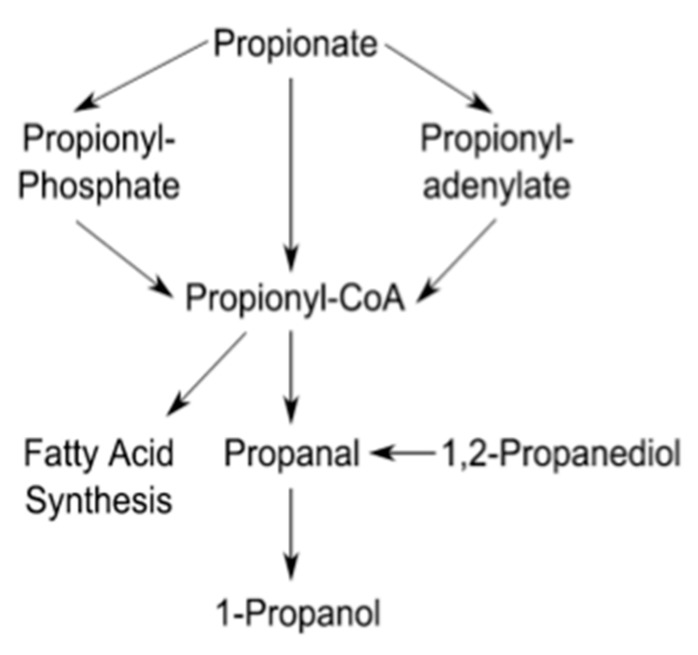
The propionate metabolic pathways based on the annotated genome. Information was retrieved from the KEGG database.

While the impact of propionate on *L. monocytogenes* carbon metabolism was observed, it was not clear whether propionate was actually metabolized under our culture conditions. According to the annotated genome, *L. monocytogenes* is capable of the synthesis of propionyl-CoA from propionate in three different pathways (Figure 5). Because propionyl-CoA can be used as a precursor substrate for straight chain fatty acid synthesis, propionate metabolism should result in an increase in straight chain fatty acids. Therefore, we analyzed the fatty acid composition of *L. monocytogenes* grown with or without propionate (Table 2). Compared to aerobic conditions, the proportion of anteiso-branched chain fatty acids (BCFAs) decreased dramatically under anaerobic conditions. In contrast, the proportions of iso-BCFAs, straight chain fatty acids, and unsaturated fatty acids were all higher under anaerobic conditions. As a result, the anteiso- to iso-BCFAs ratio as well as the BCFAs to straight chain fatty acid ratio were both dramatically reduced in anaerobically grown bacteria, compared to aerobically grown bacteria. These distinct differences suggest that fatty acid synthesis in *L. monocytogenes* is modulated by the presence or absence of oxygen. 

The presence of propionate resulted in notable increases in the proportion of odd-numbered, not even-numbered, straight chain fatty acids and a small increase in unsaturated fatty acids, accompanied by the concomitant decreases in the proportions of anteiso- and iso-branched chain fatty acids, under both aerobic and anaerobic conditions. These results agree with an earlier study investigating the effects of propionate on fatty acid composition under aerobic conditions [29] and strongly support the ability of *L. monocytogenes* to utilize propionate as a precursor for straight chain fatty acid synthesis.

### 2.5. Propionate Perturbation on L. monocytogenes LLO Production

**Figure 6:**
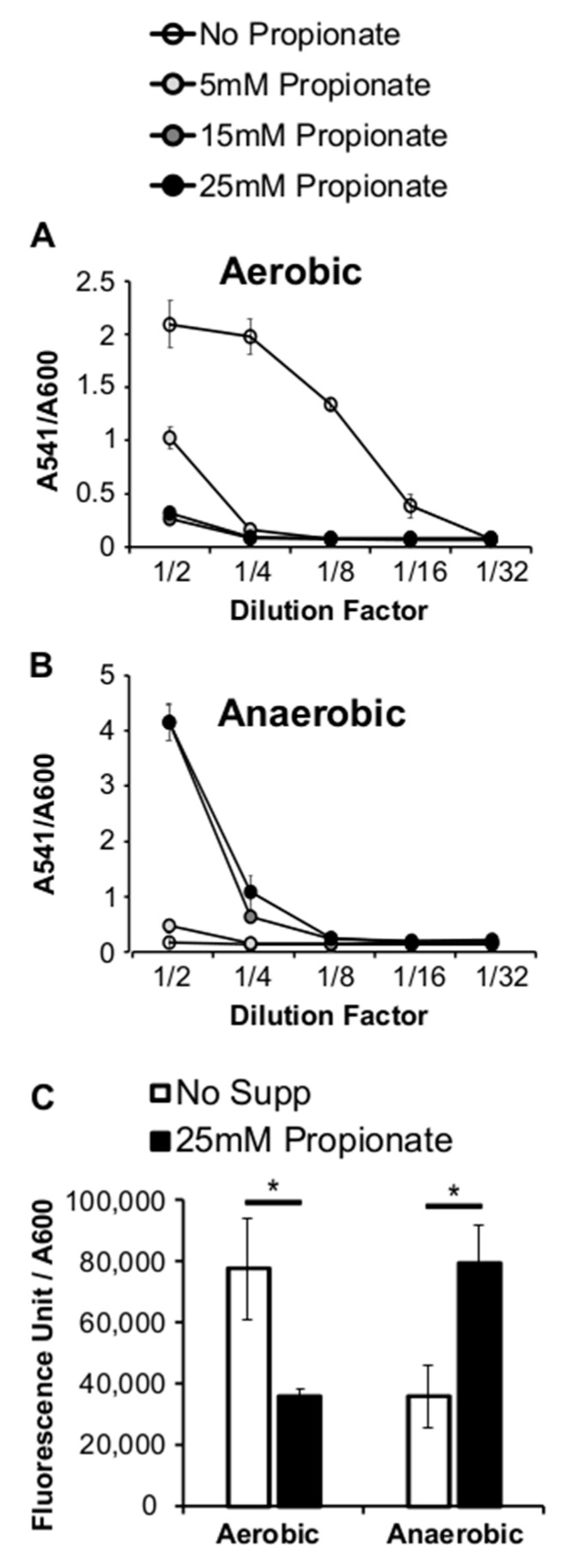
Supplementation of exogenous propionate resulted in decreased supernatant hemolytic activities under aerobic conditions (**A**) but increased supernatant hemolytic activities under anaerobic conditions (**B**). A *hly* promoter driven reporter strain, when grown aerobically, produced less fluorescence in the presence of propionate. When grown anaerobically, it produced more fluorescence in the presence of propionate (**C**). Hemolytic activities were normalized by culture optical densities measured at 600 nm. Averages of triplicates were plotted with error bars representing standard deviations. Results represent three independent experiments. “*” indicates *p* values between 0.05 and 0.01.

**Figure 7:**
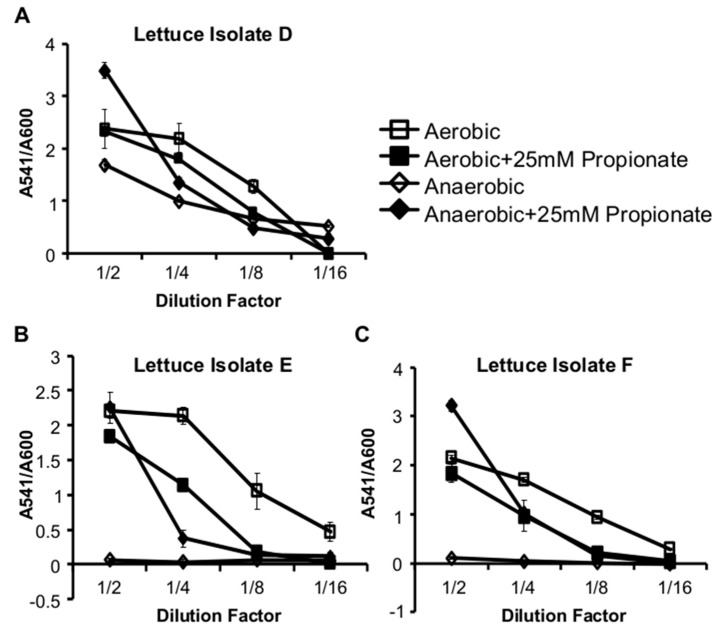
Propionate supplementation resulted in decreased supernatant hemolytic activities under aerobic conditions and increased supernatant hemolytic activities, normalized by culture optical densities, under anaerobic conditions for three environmental isolates from lettuce (**A**–**C**). Averages of triplicates were plotted with error bars representing standard deviations. Results represent three independent experiments.

The membrane fatty acids of *L. monocytogenes* are intricately involved in its pathogenesis [30,31]. The alterations in fatty acid composition caused by propionate may therefore lead to changes in virulence regulation. To determine whether *L. monocytogenes* virulence regulation is modulated by propionate, we measured the production of listeriolysin O (LLO), one of the main virulence factors of *L. monocytogenes*, in cultures grown with or without propionate. Using a standard hemolytic assay to measure the activity of secreted LLO, we noted a dramatic decrease in the culture supernatant LLO activity in aerobic cultures supplemented with propionate, compared to that in aerobic cultures without propionate (Figure 6A). In contrast, under anaerobic conditions, propionate supplementation resulted in an increase in supernatant LLO activity (Figure 6B). This phenomenon was confirmed by measuring the transcriptional activity of the *hly* promoter using a GUS reporter strain (Figure 6C), indicating that the oxygen-responsive regulation occurs at the transcriptional level. To ensure this opposing effect of propionate on LLO production was not an irrelevant phenotype observed only in the laboratory strain, we isolated several *L. monocytogenes* strains from garden lettuce and observed similar effects of propionate on LLO production in these isolates (Figure 7). These data suggest a potential impact of propionate on *L. monocytogenes* pathogenesis and further highlight that *L. monocytogenes* response to propionate was influenced by the presence or absence of oxygen. 

## 3. Discussion

Propionate is a safe and ubiquitous compound both as a natural metabolic product of our intestinal microbiota and a common food additive. The levels of propionate in the human wet fecal matter can reach as high as 185 mmol/kg [32]. As a GRAS food additive, propionate can be used at up to 3000 mg/kg [33], which is approximately 4 mmol/kg. Therefore, propionate concentrations tested in this study—up to 25 mM or 25 mmol/L—is within the realistic levels of propionate experienced by enteric pathogens such as *L. monocytogenes*. Adaptations to propionate, likely taking place under reduced oxygen conditions because of the suboxic food packaging and the anaerobic lumen in the intestines, must then be frequent events for these pathogens. However, the effects of propionate on foodborne pathogens, particularly under anaerobic conditions, are not well understood. Therefore, in this study, we investigated how the foodborne pathogen, *L. monocytogenes*, responds to propionate under both aerobic and anaerobic conditions to better understand the effects of propionate on *L. monocytogenes* fitness and pathogenesis. We reported here a notable impact of propionate on *L. monocytogenes* planktonic and adherent growth, and carbon metabolism, changing ethanol and acetoin production, as well as fatty acid composition. The production of the virulence factor, LLO, was also sensitive to regulation by propionate in an oxygen-dependent manner.

### 3.1. Propionate Perturbation on L. monocytogenes Growth

The inhibitory effects of propionate on *L. monocytogenes* planktonic growth in BHI at 37 °C are strongly influenced by pH, oxygen levels, and media buffering. The inhibitory effects of propionate at 37 °C were only observed in media buffered at pH 6.0 under aerobic conditions and in unbuffered media under anaerobic conditions. We predict that this difference is caused by the differential ability of *L. monocytogenes* to activate acid tolerance response (ATR). As a weak acid with a pKa value of 4.88 [34], the protonated form of propionate is more prevalent at lower pH and can diffuse across biological membranes. As the acid enters the neutral cytosol, it dissociates into free organic acid and protons, resulting in an acidified cytosol that can lead to stunted growth. Typically, *L. monocytogenes* possesses ATR that contributes to its survival in acidic food products [35,36,37]. It is possible that the ability of *L. monocytogenes* to activate acid tolerance response to mitigate the effects of propionate varies, depending on media buffering and oxygen levels. In addition to acid exposure, other factors, such as surface, heat shock, and osmotic shock [38,39], have also been implicated in inducing ATR. Moreover, limiting oxygen was found to activate ATR and enhance *L. monocytogenes* survival in acids [40]. Therefore, more in-depth investigations are necessary to reveal how pH buffering and oxygen levels impact the efficacy of propionate in controlling the planktonic growth of *L. monocytogenes*. 

In contrast, for solid food types or surfaces where *L. monocytogenes* adherent growth is a more prevalent concern, the lack of oxygen in combination with propionate will likely be most effective in preventing *L. monocytogenes* growth. A recent study reported that *L. monocytogenes* strain Scott A exhibited increased proportion of saturated C16 and C18 straight chain fatty acids during biofilm grown on polystyrene plates [41]. Similarly, two food isolates adhered to glass wool also showed an increased proportion of C16 and C18 straight chain fatty acids compared to the planktonic samples [42]. Both of these studies were performed under aerobic conditions and agree with our aerobic observations that aerobic propionate supplementation resulted in increased proportions of straight chain fatty acids as well as adherent growth on polystyrene plates. However, under anaerobic conditions, the enhanced adherent growth by propionate supplementations was not observed. Therefore, factors that contribute to *L. monocytogenes* adherent growth are likely regulated by the presence of absence of oxygen, independently from the regulation by the proportions of straight chain fatty acids. Given the diverse range of biofilm forming ability observed in large numbers of different *L. monocytogenes* strains [43,44,45,46], targeting membrane fatty acid composition as an environmental contamination control strategy will require a more extensive understanding of how fatty acid composition is influenced by environmental conditions in *L. monocytogenes*.

### 3.2. Propionate Perturbation on L. monocytogenes Metabolism

Based on genome annotations, *L. monocytogenes* is capable of generating propionyl-CoA from propionate through three different pathways (Figure 5). If propionate supplementation results in an enrichment of propionyl-CoA, which can be utilized as a substrate for straight chained fatty acid synthesis [47], we would expect an increase in straight chain fatty acids in propionate-treated *L. monocytogenes*. Moreover, we have demonstrated in earlier work that supplementation of butyrate, a four carbon carboxylic acid, resulted in a large increase in even numbered straight chained fatty acids [30]. Supplementation of propionate, a three carbon carboxylic acid, should therefore result in an increase in odd numbered straight chained fatty acids. Indeed, with 25 mM propionate supplementation, the proportion of odd numbered, not even numbered, straight chained fatty acids increased (Table 2). These data strongly suggest that *L. monocytogenes* is capable of directly metabolizing propionate as a precursor for the synthesis of odd numbered straight chain fatty acids—an ability likely enhanced under anaerobic conditions. The phosphotransbutyrylase, encoded by the *ptb* gene, is capable of liberating Coenzyme A from propionyl-CoA with a *K_M_* of 190.6 ± 20.9 µM and *k_cat_*/*K_M_* of 2.72 (µM^−1^ s^−1^) [48]. While the phosphotransbutyrylase may be involved in incorporating propionate into odd numbered straight chain fatty acids, whether its expression or activity is regulated by the presence or absence of oxygen is unclear.

Moreover, although the increase in odd numbered straight chain fatty acids was observed under both aerobic and anaerobic conditions, the extent by which propionate supplementation alters the ratios between anteiso- and iso-branched chain fatty acids were different between aerobically and anaerobically grown *L. monocytogenes*. Propionate supplementations did not alter the anteiso- to iso-branched chain fatty acid ratios under aerobic conditions but raised the ratio in anaerobically grown *L. monocytogenes*, an observation suggesting that the ability of *L. monocytogenes* to synthesize specific subtypes of branched chain fatty acids is impacted by propionate under anaerobic but not aerobic conditions. FabH, the enzyme that facilitates the incorporation of acyl-CoA substrates into the fatty acid synthesis pathway, exhibits a selectivity toward branched acyl-CoA over straight acyl-CoA, resulting in a membrane composition enriched with branched chain fatty acids [49]. Moreover, among different branched acyl-CoA, the preference for the precursors for anteiso-branched chain fatty acids was also enhanced at 10 °C, compared to 30 °C [50]. Whether the substrate preference of FabH is regulated by oxygen or by propionate is also unclear. Nevertheless, our data introduce oxygen level as a key factor in how *L. monocytogenes* modulates membrane fatty acid composition in response to environmental conditions.

In addition to the positive impact on the proportion of straight chain fatty acids, supplementation of propionate also led to a significant increase in acetoin production under aerobic but not anaerobic conditions. Acetoin production in *L. monocytogenes* typically takes place under aerobic conditions [51,52] but can be stimulated under both aerobic and anaerobic conditions if exogenous pyruvate, the substrate for acetoin production, is introduced [53]. Therefore, based on our results, the regulation of acetoin production by propionate likely occurs downstream to the regulation by oxygen. Earlier studies have shown that deficiency in the synthesis of aromatic compounds [52] as well as exogenous lactate and diacetate [54] both led to an increase in aerobic production of acetoin. Whether propionate supplementations, and the associated alterations in membrane fatty acid compositions, share a similar signaling pathway as aromatic deficiency or lactate and diacetate treatments remains to be determined.

### 3.3. Propionate Perturbation on L. monocytogenes Pathogenesis

*L. monocytogenes* virulence regulation is intricately linked to its carbon metabolism. The activity of the master virulence regulator PrfA, which is involved in the activation of LLO production, is regulated by available carbon sources [55,56,57]. The PrfA regulon, in return, also includes genes involved in carbon metabolism [58]. Numerous studies have provided clues on *L. monocytogenes* metabolism inside host cell cytosol [59,60,61], indicating the flexibility of *L. monocytogenes* carbon metabolism in response to different environmental conditions inside and outside of host cells. It is known that one of the environmental factors that controls *L. monocytogenes* carbon metabolism is the presence or absence of oxygen [51,60], with higher ethanol and lactate production under anaerobic conditions and higher acetoin production under aerobic conditions. The perturbations in alcohol and acetoin productions by propionate supplementation suggest that despite the limited genome annotations in pathways directly involving propionate, the impact of propionate exposure on *L. monocytogenes* carbon metabolism may be more extensive than anticipated. Whether the metabolic effects of propionate on *L. monocytogenes* contribute to virulence is currently not known. However, if *L. monocytogenes* is in frequent contact with propionate, especially in the anaerobic lumen of the human intestines, its adaptations to propionate may influence its subsequent infections inside the host cells.

In conclusion, we reported here that environmental exposure to propionate alters *L. monocytogenes* planktonic and adherent growth, carbon metabolism, and LLO production. Moreover, the effects of propionate on *L. monocytogenes* are strongly modulated by pH, temperature, and the presence or absence of oxygen. To prevent *L. monocytogenes* growth during food processing and storage, all these factors—pH, temperature, oxygen levels, and food matrix types, contribute to the efficacy of propionate. The aerobic suppression and anaerobic induction of LLO production in response to propionate exposure, observed both in our laboratory strain 10403s as well as environmental isolates, highlight the relevance of our findings and the potential for propionate exposure by *L. monocytogenes* to alter subsequent interactions with host cells.

## 4. Materials and Methods

### 4.1. Bacterial Strains and Culture Conditions

The wildtype *Listeria monocytogenes* strain 10403s (serotype 1/2a) and an isogenic reporter strain (P*_hly_-gus-neo*) [56] were used in this study. Additional environmental isolates of *L. monocytogenes* used in this study were cultured from lettuce samples described below. For all experiments, *L. monocytogenes* was grown overnight in filter-sterilized brain heart infusion (BHI) media. Buffered BHI was made with 100 mM sodium monobasic and dibasic phosphate salts. For aerobic growth, bacteria were grown in a 37 °C incubator or at room temperature with shaking at 250 rpm. For anaerobic growth, bacteria were grown statically a 37 °C in a temperature-controlled incubator or at room temperature in an anaerobic chamber (Type A, Coy Laboratory, Grass Lake, MI, USA). The chamber contains a nitrogenous atmosphere with 2.5% hydrogen. Optical density (OD) was measured in a 96-well plate at 600 nm with a volume of 200 μL per well using a 96-well plate reader (Synergy4, Biotek, Winooski, VT, USA). Sodium propionate stock solutions (A11148, Alfa Aesar, Haverhill, MA, USA) were prepared at 1 M in deionized water, filter-sterilized, and stored in the −20 °C freezer. 

### 4.2. Isolation of Listeria from Garden Vegetables

A sample of garden lettuce (25 g) from a local organic garden was blended with 200 mL of Buffered *Listeria* Enrichment Broth [62] for 30 s. The suspension was serially diluted (1:10) and incubated at 30 °C for 4 h. Three different antibiotics were then added to all tubes: cyclohexamide, naldixic acid, and acriflavin [63]. The tubes were incubated at 30 °C for 48 h. After incubation, the bacterial suspensions were streaked onto Modified Oxford Agar (#R01613, Fisher Scientific, Hampton, NH, USA) and Brilliance *Listeria* agar plates (CM1080B and SR0227E, Fisher Scientific). Plates were placed in a 37 °C incubator for 48 h to select for *L. monocytogenes* growth. Colonies that grew were chosen at random and streaked onto BHI plates and again incubated at 37 °C for approximately 48 h. Identities of colonies that grew on BHI plates were confirmed (data not shown) by qPCR using primer sets unique for *L. monocytogenes* (Forward: 5′-AACTGGTTTCGTTAACGGTAAATACTTA; Reverse: 5′-TAGGCGCAGGTGTAGTTGCT) and general for all *Listeria* species (Forward: 5′-GTTAAAAGCGGTGACACTATTTGG; Reverse: 5′-TTTGACCTACATAAATAGAAGAAGAAGATAA) [64].

### 4.3. Adherent Growth Assay

Adherent growth assay was performed by growing cultures from fresh colonies in sterile polystyrene 96-well plates at 100 μL per well for 24 h. After cultures were removed, wells were washed five times with water and dried for 30 min. To stain the adherent bacteria, crystal violet (1% w/v in water, 150 μL per well) was added to each well. After 45 min of staining, plates were washed five times with water and dried for 30 min. Ethanol (95%, 200 μL per well) was added to each well to solubilize the stain. Absorbance of the dissolved crystal violet was read at 595 nm to represent with the amount of adherent bacteria.

### 4.4. Measurement of Acetoin, Ethanol, and Lactate Concentrations

Acetoin production in the supernatant of overnight *L. monocytogenes* cultures was quantified by an adapted Voges-Proskauer test. Briefly, 100 μL of culture supernatant sample or acetoin standard was mixed with 50 μL of 5% creatine monohydrate (C3630-100G, Sigma, St. Louis, MO, USA), 100 μL of 5% 1-Napthol in 95% EtOH (N1000-10G, Sigma), and 100 μL of 40% KOH in 95% EtOH. The mixtures were incubated for 15 min at room temperature prior to absorbance reading at 560 nm. Based on the standard curve created from acetoin samples of known concentrations, the concentration of acetoin in culture supernatant samples was calculated. Percentage of ethanol in culture supernatant was determined using a commercially-available ethanol assay kit following the manufacturer’s suggested protocol (Fisher 50-489-254). Lactate production in the supernatant was quantified by a colorimetric assay [65] in which 50 μL of supernatant sample or lactate standard was mixed with 300 μL of concentrated sulfuric acid, 5 μL of 4% cupric sulfate, and 10 μL of 1.5% *p*-phenylphenol (in 95% ethanol). The mixtures were incubated at room temperature for 15 min prior to absorbance reading at 570 nm. Standard curves of lactate dissolved in BHI were used to determine the concentration of lactate in the culture supernatant samples.

### 4.5. FAME Analysis

Bacterial cultures (15 mL) were grown overnight in BHI supplemented with or without 25 mM sodium propionate under aerobic and anaerobic conditions. Bacteria were harvested by centrifugation (10,000 rpm, 10 min), washed once with PBS, and frozen in an ethanol-dry ice bath. Frozen pellets were shipped to Microbial ID Inc. for Direct FAME (fatty acid methyl esters) analysis with gas chromatography.

### 4.6. Hemolytic Assay

Hemolytic assays were performed using the supernatant from *L. monocytogenes* overnight cultures. Samples (100 μL) were added to wells with 5 μL 0.1 M DTT and incubated at room temperature for 15 min. Blank BHI medium was used as a negative control, and 0.4% Triton X-100 was used as a positive control for each experiment. Samples were serially diluted (1:2) using hemolysis buffer (35 mM dibasic sodium phosphate, 125 mM sodium chloride, brought to pH 5.5 using acetic acid). Defibrinated sheep’s blood (DSB050, Hemostat Laboratories, Dixon, CA, USA) was diluted to a hematocrit of 2% in hemolysis buffer and added to samples to achieve 1% final hematocrit. Samples were incubated at 37 °C for 30 min and centrifuged to pellet intact cells. Supernatant lysate (120 μL) was placed into a flat-bottom 96-well plate for absorbance reading at 541 nm as an indicator for LLO activity. 

### 4.7. MUG Assay

The *L. monocytogenes* reporter strain (P*hly-gus-neo*) was generously provided by Dr. Nancy Freitag at University of Illinois College of Medicine at Chicago to establish transcriptional responses of *hly* to propionate. The reporter strain was grown on LB plates with neomycin sulfate (1 µg/mL). Colonies were selected and used to inoculate into BHI with or without propionate for growth overnight. Optical density (OD) of the overnight cultures was measured for normalization. The bacteria (1 mL) were harvested by centrifugation at 4 °C, washed twice with PBS, and resuspended in 100 μL of PBS with 1% Triton-X100. Bacterial cells were then lysed using a sonicator for three 30-s cycles. Samples were put on ice between each cycle. The lysate samples were centrifuged at 10,000 rpm for 5 min at 4 °C, and the resulting 100 μL of supernatant was collected into a 96-well plate. In the dark, 20 μL of 4-Methylumbelliferyl-β-d-glucuronide solution (1.8 mg/mL MUG; AAB21190MD, Fisher Scientific) was added to each well, and the plate was incubated at 37 °C. After 10 min, 10 μL of 0.2 M sodium carbonate was added as a stop solution in the dark. Fluorescence was measured at 365 nm excitation wavelength and 400 nm emission wavelength using a 96-well plate reader (Synergy4, Biotek). 

### 4.8. Statistics 

Statistical analyses were done in Microsoft Excel with *p*-values between each pairwise comparison calculated by two-tailed Student’s *t*-tests. 

## Figures and Tables

**Table 1:** Effects of exogenous propionate on *L. monocytogenes* planktonic growth in BHI. Data shown here are averages of triplicates and represent at least two independent experiments. Asterisks denote significance difference between controls without propionate and samples with propionate. *, 0.05 > *p* > 0.01; **, 0.01 > *p* > 0.001; ***, *p* < 0.001.

	Aerobic	Aerobic +25 mM Propionate	Anaerobic	Anaerobic +25 mM Propionate
Doubling Time (Minutes) at 37 °C	78.06 ± 5.05	85.22 ± 4.83	73.51 ± 0.53	86.37 ± 4.96 *
Doubling Time (Minutes) at Room Temperature	170.60 ± 0.56	157.66 ± 2.11 **	119.48 ± 5.48	123.64 ± 10.84
Culture pH after Overnight Growth at 37 °C	5.28 ± 0.02	5.57 ± 0.01 ***	4.90 ± 0.02	5.29 ± 0.01 ***

**Table 2:** Fatty acid composition (in percentages) in *L. monocytogenes* grown in BHI at 37 °C under different conditions. Data shown here are averages of two independent experiments.

	Aerobic	Aerobic +25 mM Propionate	Anaerobic	Anaerobic +25 mM Propionate
15:0 Anteiso	46.24	42.15	35.65	31.8
17:0 Anteiso	36.92	26.8	24.35	12.27
**Anteiso Total**	**83.16**	**68.95**	**60**	**44.07**
14:0 Iso	0.41	0.5	0.89	0.74
15:0 Iso	9.53	7.96	11.76	5.97
16:0 Iso	2.19	1.92	3.21	1.33
17:0 Iso	3.28	2.38	4.78	1.4
**Iso Total**	**15.41**	**12.76**	**20.64**	**9.44**
**Anteiso: Iso Ratio**	**5.40**	**5.40**	**2.90**	**4.67**
13 straight	0	0.95	0	1.71
15 straight	0	14.27	0	22.87
16 straight	0.66	0.65	2.21	1.81
17 straight	0	1.38	0	2.23
18 straight	0	0.16	0.44	0.59
**Straight Total**	**0.66**	**17.41**	**2.65**	**29.21**
**Branched: Straight Ratio**	**149.35**	**4.69**	**30.43**	**1.83**
18:1 w9c	0	0.21	6.18	7.4
18:2 w6,9c	0	0.21	1.72	1.88
**Unsaturated Total**	**0**	**0.42**	**7.9**	**9.28**
